# Signs of Fascism Rising: A European Americanist Looks at Recent Political Trends in the U.S. and Europe

**DOI:** 10.1007/s12115-017-0128-7

**Published:** 2017-06-26

**Authors:** Rob Kroes

**Affiliations:** 13 Hertenlaan, 3951AS Maarn, The Netherlands

**Keywords:** Fascism, Populism, Comparative democratic systems, Corporatism

## Abstract

This paper's central concern is with signs of fascism in recent political developments in a number of European countries and the United States. It takes the reader back to earlier periods in European and American history when this same anguished question was raised. Thus a longer intellectual history of concerns about the viability of democratic systems is drawn to guide us in our current political evaluations.

As the world awoke to the news of Donald Trump’s upset victory in the American presidential election, a widely-shared feeling was of witnessing the effects of an iconoclasm, of a breaking of images at the hands of an insurgent mob. Never mind the election victory being narrow. Trump had lost the popular vote by close to three million, and won the electoral college by a mere 77.000 votes decisively cast in three mid-Western swing states. The electoral college, originally intended as one further hurdle on the way to mob rule, now had produced the opposite effect and allowed the angry voice of an insurgent minority to drown out all others. If images had been smashed in the process, they were crucially images of America, mental constructs of a country and a culture held by individuals the world over. And one need not have been a member of the international community of American Studies scholars to feel aghast at what had been done to one’s cherished inner image of an inspirational America, brought back so vociferously by Barack Obama in his meteoric passage across the skies of American politics.

The Blogosphere, in the days and weeks following Trump’s election win, resounded with impassioned comments, ranging from eloquent elegies for what had vanished to angered anticipations of what a Trump presidency had in store. What sort of a man are we dealing with? Beyond instant genteel reactions to the man seen as unspeakably vulgar, as the new president of Vulgaria, the intellectual distance provided by the academic perspective of, e.g., an American Studies tradition allows us to tap into a reservoir of American history, replete with possible precursors to present-day Trump. If beyond condescension and derision we wish to make sense of the man by placing him alongside American character types, our question should be: Is Trump more than, in Mitt Romney’s words, a fraud and a phony?^1^ Can we see him as an American original? And if so, in what sense? In the commercial sense in which a pair of Levy’s blue jeans is advertised as an American original, or in the sense in which, for instance, Mark Twain, changing the terms of cultural appreciation, was one. Is Trump a true contemporary vernacular hero in the great American tradition?

As Jonathan Raban, British writer and long-time U.S. resident reminds us^2^: “All modern American literature comes out of one book from Mark Twain called Huckleberry Finn. From Huck, via Hemingway to J. D. Salinger’s Holden Caulfield and beyond, the vernacular narrator has unmasked the pretensions and hypocrisy of the society around him by his plain speaking. Mr. Trump’s vernacular may be an unholy tangle of lies, misapprehensions, disinformation and personal insults, but it exposed Mrs. Clinton’s campaign rhetoric as stilted and artificial. She spoke in the reasoning language of the high school classroom; he did locker-room trash talk.” Trump has great gifts in the art of vengeance and humiliation. It is a ribald form of wit that can make voters laugh with the scornful jeer that, for a moment, irons out the inequalities between the 1% and the rest of the nation. In contrast to Trump’s sure sense of his audience’s wave length, Clinton proved amazingly tone-deaf, disparaging Trump supporters as “a basket of deplorables.” As the deplorables noticed, she would rather address a basket of bankers and be paid truly indecent speaker’s fees.

At best, our attempt at placing Trump as a public character takes us into an American rogue gallery, where he could hold his own alongside the conman, the snake oil vendor, the side show barker on the fairground, if not the Wizard of Oz, at work in his illusionist’s world of smoke and mirrors. These are the more picaresque, we might say P.T. Barnum-like, associations that come to mind. But there are many more of a darker hue. Throughout the period of the electoral campaign these kept popping up in the media, on both sides of the Atlantic, as they will most likely keep doing in months and years to come.

One highly apposite association was disseminated by *Public Seminar*, a group that sees itself working in the spirit of The New School for Social Research’s University in Exile and its legendary General Seminar. The original version was set up to receive scholars in exile, who came to The New School fleeing Nazi and Fascist repression. Continuing in this spirit *Public Seminar* takes up issues of vital interest in politics and society. Thus, the advent of Donald Trump on to the political stage was the occasion for raising larger issues concerning Trump’s style of leadership.^3^ After Trump had retweeted an Italian saying made famous by the Fascist dictator Benito Mussolini, this had led one American historian, an expert of Italian Fascism, to liken Trump, Berlusconi, and Mussolini under the category of “charismatic personalities,” all embodying a very similar “cult of power.” But as the *Public Seminar* authors emphasize, Trump in no way can be seen to measure up to Max Weber’s concept of charismatic leadership as developed in Weber’s famous talk, *Politics as a Vocation* (1919). As Weber argued, one prerequisite for charismatic leadership is responsibility, involving a duty to truthfulness, to speak the truth even when it is not pleasing. Also, the charismatic person must control a negative impulse that can easily tempt the political leader: vanity. So, at best, we are dealing with what American historian Daniel Boorstin would have called pseudo-charisma, a fake version of the real thing. If not charisma, what then can be taken to account for Trump’s public appeal? A more likely candidate would be the trickster, a Jungian archetype stalking through the human imagination and cultural memory in – as is his wont - many forms and guises. The trickster knows no bounds; these only exist for him to trespass, construct anew, and in the end to destroy. If he knows joy, it is the nihilist’s joy of destruction, or better, his infatuation with destruction.^4^ Or as Michael Sakamoto, in his study of Japanese Butoh dance theater, puts it: „Tricksters essentialize change. They are amoral and behave as incoherently as they please. They are not (or do not consider themselves) beholden to deities or any rules of consistent behavior, real circumstances being their only mandate for action. Almost by definition, they adopt different personae as situations call for them. “^5^ If this is beginning to typify Trump for us, it may also help us place Trump among kindred types.

But do we have to plumb the depths of a Jungian collective subconscious to make sense of Trump as a public phenomenon? There is also, more specifically, collective memory to guide us. And there we have a rich reservoir of historical precursors and prototypes. Thus, the German weekly newspaper *Die Zeit*, on October 27th, 2016, had a full-page essay, by leading German journalist and political commentator Josef Joffe, entitled “Amerika’s blonder Mussolini (America’s blond Mussolini).” In it, Joffe connects the Trump phenomenon to similar movements in Europe, and points to the common element they all share: the enraged citizen, a term that translates well across national borders, from German “Wutbürger,” to French “enragés,” or Spanish “indignados.” Together they form the rank and file of the insurgent movements that threaten to explode the established bounds of democratic politics. They feel no longer duly attended to by the ways of party politics and representational democracy. They feel left out and ignored, having to fend for themselves in the face of larger transformative social forces, such as immigration and globalization. All manner of accustomed social and economic balances, of race and ethnicity, of relative social rank, of economic security, have been tilting for years, if not decades. They constitute shifts that have affected people differentially, benefiting a minority, while holding back the others.

But Joffe does more. Not only does he point to similar trends and developments as they occur today, on both sides of the Atlantic, he also points to historical antecedents. Calling Trump a blond Mussolini, Joffe - and many other observers of current politics – recognize the features of fascism as first propagated by Mussolini in Italy. At the time, in the early twentieth century, Mussolini was among those, intellectuals and political activists, on the political left and right, who were searching for alternatives to liberal democracy. As they saw it, liberal democracy, as shaped under bourgeois auspices in the nineteenth century was a set of institutional arrangements for harnessing the political forces unleashed by enlightenment ideas of individual citizens forming the central actors in a democracy. As Mussolini and other critics saw it, the expression of the people’s will, through elections, political parties, and parliaments, was doomed to fail. It would never be more than a headcount of individual votes, and never transcend that level of hyper-individualism. It could never speak in the voice of the meaningful collectivities in which people lived their lives. Particularly with the advent of the general franchise, and the advent of “the masses,” as they were disparagingly called at the time, the political arena splintered, unable to cope with the inchoate jumble of voices. Of course, liberal democracy is much more than just a matter of single individuals casting their individual votes every so many years. That would be a caricature, but one used to great effect by the many enemies of democracy.

Over a century ago authoritarian movements, out to impose their will on existing democracies, saw fit to sabotage and delegitimize the institutional apparatus of democracy. They bullied their way on to the political agora through intimidation, the threat of violence or its actual use. They undermined the citizen’s faith in the rule of law, the public media and the representative system. Today’s populists are working the same vein. They call the workings of the democratic system rigged, while actively doing the rigging themselves. Think of the way the Republican Party in the U.S. saw it as its main mission to block any initiative or program identified with the Obama administration.^6^ They cast aspersions on the fourth estate, the press, while subverting the very idea of a free exchange of opinion as the lifeblood of a deliberative democracy. Crucially they reduce factual truth to matters of mere opinion. They ridicule the rule of law through disparaging the courts, depicting them as the servants of special interests only. Small wonder that so many people have lost trust in representative bodies like parliaments, in political parties and political leaders, or more generally in democratic politics. It takes a special, almost nihilist, willingness to engage in the willful destruction of democratic ways, political rights and legal protections to allow a new world order to be borne, a come-what-may belief in purifying and regenerative violence. Yet that is what drove a man like Mussolini, inspired by ideas in vogue at the time, in left-wing syndicalist circles - think of George Sorel’s almost cinematic evocation of the final, world-changing general strike – or in right-wing authoritarian milieus, in France and elsewhere in Europe. Amazingly, the appeal of regenerative violence was also current in circles of economic theoreticians – think of Joseph Schumpeter, and his ideas of the impact of capitalism as a matter of creative destruction – or in avant-garde circles in the arts.

Today one must be truly angered to become infatuated with dreams of utter destruction, yet surprisingly these sentiments have been shown to exist, particularly among Trump supporters. Trying to establish what drives people who have become disaffected from politics and are casting about for alternatives, Douglas Rivers, researcher for YouGov, found this: asked whether his respondents would rather have a president who radically overhauls the country from the bottom up, even when it might make things worse, eight out of ten Trump supporters say “yes,” against 2 out of ten among Clinton supporters. As Laura Stoker, political scientist at Berkeley, sums it up: only the truly incensed and insurgent would take the risk, feeling as fooled by the system as they do.^7^ Amazingly, these destructive sentiments are also being held by people as high up in the Trump hierarchy – the Trump tower of power, so to speak – as Steve Bannon. This is what he is reported to have said in 2013: “I’m a Leninist,” Bannon proudly proclaimed. Asked what he meant, he answered: “Lenin wanted to destroy the state, and that’s my goal too. I want to bring everything crashing down, and destroy all of today’s establishment.” Bannon was employing Lenin’s strategy for Tea Party populist goals. He included in that group the Republican and Democratic Parties, as well as the traditional conservative press. His goal was to bring down the entire establishment including the leaders of the Republican Party in Congress.^8^

This may be a moment of history repeating itself. The appeal of anti-democratic demagogues across western nations does in fact look like a throwback to the times around the turn of the twentieth century. What had only been stirrings of anti-Enlightenment sentiment up to then, would fully come into its own as a full-blown attack on liberal democracy. By then politics had become mass politics and the anti-Enlightenment could inspire a mass movement, Fascism. But the rise of the latter was only possible because the liberal bourgeoisie had not succeeded in creating a new spiritual base, in providing the people with a common myth and a feeling of being in the service of some higher cause rather than their personal interests. Democracy itself came to be regarded as an evil: it stood accused of conceiving society as a mere aggregate of individuals, without a shared sustaining myth, without emotional solidarity.

This void at the center fascism set out to fill with visions of the organic unity of the nation, of the history it shared, of the destiny it was promised. And the embodiment of this unifying vision was to be in the leader, the Duce, the Führer, who in mythical union with the people would be able to express their common purpose, unmediated, in cinematic rituals of communion with the masses or in post-modern tweet-storms as a virtual version of direct communion.^9^ Fascism, thus, in its claims of universal, if not – as Mussolini liked to have it – totalitarian embodiment of the organic unity of the nation, was neither right nor left. In its anti-liberal rebellion, it had a strong appeal among working-class people while fascinating a great many intellectuals, in Europe and the United States, people like Benedetto Croce, Gaetano Mosca, Charles Beard, and Robert Michels.^10^ Thus, the latter, famous student of political party systems, whose name had forever become linked to the “iron law of oligarchy,” to the idea that organizations, no matter how democratic in their original design and intent, unavoidably declined into oligarchies, had words of praise for Mussolini’s experiment. In a 1928 Cologne lecture, in which he compared the economic and political systems of Italy and the United States, he saw rejuvenation and revitalization in Mussolini’s fascist state. Having done away with political parties, freedom of the press and parliamentarism as roadblocks on the way to the fascist state Mussolini’s fascist revolution had delivered a new form incorporating all of society’s diversity.

In America on the other hand he saw his forces of oligarchization at work, undermining democratic life. In the rise of “machine politics,” political corruption and low turnout even among groups newly enfranchised, Michels recognized signs of America’s democracy losing its claims to being representative.^11^ Karl Mannheim, a contemporary of Michels, and internationally known observer of the upheaval brought by the advent of mass society, also saw the totalitarian movements of his time as forces doing away with the forms of liberal democracy. As late as 1931 he could still make light of Hitler’s movement, assuring the American journalist Dorothy Thompson that Hitler was “just a clown.” Shortly after he had found refuge in England, having witnessed the collapse of the Weimar Republic and the demise of liberal democracy in Germany. Interestingly, the experience of political life in England revived his spirits. Witnessing the continued pulse and energy of democratic life suggested to him an alternative view of democracy. England showed him an alternative to what he and like-minded intellectuals had critiqued in the unhinged mass democracies of Germany and Italy. In England, political representation organized in a system of political districts was not just a matter of individual heads being counted on a national basis, but of communal entities expressing themselves in a common voice. England was close to what fascism had been looking for, a system of corporate entities – in this case individual parliamentary districts - able to give aggregate expression to communal bonds. In a book written from his new British vantage point – *Diagnosis of Our Time* - he could now see the Nazi group strategy as the systematic disorganization of society while resting assured that this was not the sole, fore-ordained future of democracy.^12^ There is irony in the fact that in the United States the congressional district system, combined with its electoral college, can work to give regionally concentrated collective demands – those feeling left behind, the insurgent minorities – added weight, tilting the over-all electoral vote the other way. Mussolini might have liked this aspect of corporatist representation, Trump certainly did. There is further, cruel, irony in the fact that just recently the U.K. has resorted to a tool, quasi-democratic, but essentially at odds with its established tools of representative democracy – the referendum – to wreak havoc on a scale as had been inconceivable so far. The referendum opened the floodgates to a reservoir of pent-up anger, without mediating institutions, like a wrecking ball that would have pleased the likes of Mussolini.

As for the United States, “In many respects,” writes journalist Jonah Goldberg, “the founding fathers of modern liberalism, the men and women who laid the intellectual groundwork of the New Deal and the welfare state, thought that fascism sounded like ... a worthwhile 'experiment'.”^13^ The degree to which progressive and fascist values complemented and echoed one another was on clear display in the work of the progressive writer and *New Republic* founder Herbert Croly. The journalist J. T. Flynn – perhaps the best-known anti-FDR muckraker of the 1930s - foresaw that American fascism might one day manifest itself as “a very genteel and dainty and pleasant form of fascism which cannot be called fascism at all because it will be so virtuous and polite.”

Many of Mussolini’s early supporters turned away in disgust once fascism began to show its ugly face of repression and persecution of fascism’s enemies, a face more clearly to be seen shortly after in Hitler’s version of totalitarianism. But it took many people time to change their perceptions. Dorothy Thompson, celebrated American journalist working in Germany, had judged Hitler a man of “startling insignificance,” when meeting him in 1931, but realized her mistake by mid-decade: “No people ever recognize their dictator in advance,” she reflected in 1935. “He never stands for election on the platform of dictatorship. He always represents himself as the instrument (of) the Incorporated National Will.” Applying the lesson to the US, she wrote: “When our dictator turns up you can depend on it that he will be one of the boys, and he will stand for everything traditionally American.”^14^ It is easy now to see all these admonitions as strangely prescient, and to see Trump’s facial features pop up before our eyes, yet a prevailing sense in 1930s’ America was that America’s democracy would stay the course. There were voices and noises from left and right, yet they never drowned out Roosevelt’s reassuring mastery of political communication. Alternative outcomes had so far, fortunately, remained the stuff of nightmarish phantasies.

“It can’t happen here? My friends, it *is* happening here.” So says New York Mayor Fiorello LaGuardia near the end of *The Plot Against America*, Philip Roth’s 2004 novel in which he re-imagines American history if the isolationist, Hitler-sympathizing anti-Semite Charles Lindbergh had become president instead of Franklin Roosevelt.^15^ And so “the right-wing saboteurs of democracy – the so-called patriots and the so-called Christians” of the Republican right, Roth writes, make their march “under the sign of the cross and the flag.” In its depiction of the gradual, step-by-step establishment of an authoritarian, and anti-Semitic, regime, the novel takes its cue from the way Hitler consolidated totalitarian control in 1930s’ Germany. Focusing on the way the Jewish population was slowly but relentlessly cast outside civil society until the seismic events of Kristallnacht, the night of the breaking glass, in 1938 – unforgettably depicted in Sebastian Haffner’s *Germany: Jekyll and Hyde* - Roth’s book transplants the story to America. He shows how fear enters the lives of entire Jewish neighborhoods as their bonds to the larger society get severed relentlessly.

“It can’t happen here” were words of a special resonance in the 1930s. It was like a mantra for Americans to re-assure themselves in the face of the political cataclysms befalling European countries. It is also the title of Sinclair Lewis’s 1935 novel that imagined a fascist senator who beats Roosevelt to the presidency on a promise – surprise, surprise – to make America great again: “To you and you only I look for help to make America a proud, rich land again,” the imaginary Buzz Windrip tells a crowd that includes his paramilitary “Minute Men,” who beat up dissenters and open fire on them. Borrowing leaves from various rivals to Roosevelt’s New Deal, like the Townsend plan to give every citizen a free pension – a plan that aroused unexpected and wide-spread support – Windrip wins the elections. Once in power it is not long before he reneges on promises made and stamps down on enthusiasms raised. Nor did it take long for a full-fledged police state to be in place, with concentration camps locking up political opponents, and political thugs controlling the streets. Lewis was acutely aware of the political turmoil in the United States; in addition, he had intimate knowledge of the rise of fascism in Hitler Germany through Dorothy Thompson, his then-wife, who had been expelled from Germany by the Nazis in 1934. The protagonist of Lewis’s novel is an old-time, small-town newspaper editor, Doremus Jessup, who had said in 1935, in a spirit of American exceptionalism: “If there ever is a Fascist dictatorship here, American humor and pioneer independence are so marked that it will be absolutely different from anything in Europe.” (p 284) Having learned the hard way, through underground resistance work, through imprisonment, through reading books that now had become banned by the new regime, he had won a new insight into the coming and working of Fascist dictatorship. He had begun to see “something like a biology of dictatorships, all dictatorships.” (p. 285) “All dictators followed the same routine of torture, as if they had all read the same manual of sadistic etiquette.” (A manual by the way that is once again eagerly taken up by Donald Trump during the presidential campaign^16^) “And now, in the humorous, friendly, happy-go-lucky land of Mark Twain, Doremus saw the homicidal maniacs having just as good a time as they had had in central Europe.” (p.285) If Lewis’s point, calling his book *It Can’t Happen Here*, had been that not just “It might”, but in fact “It could happen here,” it is that immediacy of the threat that is brought home again with the rise of Trump. As Alain Frachon, a French chroniqueur writing for the French daily newspaper *Le Monde*, put it as early as March 19th, 2016, referring to Lewis’s novel: “Windrip has remained a fictional character. A mere few weeks ago, experts judged a victory of Trump in the Republican primaries to be a matter of pure silliness. Today, Trumpism is a political reality.”^17^ Nor was the rise of Trump the only moment reminding us of the prescient power of Lewis’s novel. There had been moments before, especially in the years of senator Joseph McCarthy’s anti-communist witch hunt in the late 1940s, early 1950s, that hammered home the dangers of a spirit of persecution descending upon the U.S. The chilling images of McCarthy’s Senate hearings, reminiscent of Stalin’s 1930s’ show trials – as brought back in Arthur Koestler’s widely read novel *Darkness at Noon* – gave a face to the era, with the features of the senator alongside his obsequious crony Roy Cohn. As it happened, the latter came back many years later as one of Donald Trump’s legal advisors, but also as the man who taught the young Donald Trump the tricks of his bruising and bullying style.

Much as Trump may stand in a line of toxic descent in American history, a line connecting the outbursts of nativism and nationalism, of racism and white supremacy, of hatred of strangers and social and cultural diversity, there is nothing re-assuring in the idea that “we have been here before.” For indeed, never so far has the line curved upwards as steeply as it has now, reaching the level of the highest governmental bodies of the American Republic, the presidency, the Congress, the Supreme Court. Following a previous peak, in the early 1960s, American historian Richard Hofstadter could still suggest that what he called the “pseudo-conservative style,” as displayed in the McCarthy years, was one of the long waves of twentieth-century American history, and not a one-off, momentary mood.^18^

But now we can’t be so sure. What with one of the two great political parties supinely shifting into surrender mode, with a new president continuing the viciousness of his campaign rhetoric in his inauguration speech, using words like carnage to describe the havoc wreaked by the preceding administration,^19^ and – most ominously – what with a post-election Alt-Right meeting in a federal building in Washington D.C. shouting “Heil the People, Heil Victory,”^20^ the president is like a spearhead penetrating into the political system, followed by the angry cohorts that see in him their leader. When he angrily promises never to let those down that feel left behind, never mind that at no point in his life has he done anything to give credence to his promise. Rather he surfs the wave of popular discontent to carry him into politics. It is like a cruel inversion of John Kennedy’s inaugural call never to ask what the country can do for you but what you can do for the country. Trump as a leader had no language for bringing back unity to a country deeply divided over his person and style of leadership. Trump had nothing to offer those outside the circle of his followers; instead he renewed their feelings of resentment, their bitter grievances, their feelings of having been “conned.” No man, ironically, more expert in those matters than Mr. Trump. Nor did he offer wider perspectives on policy areas that as President he must cover. No president-elect in modern American history has talked less about America’s obligations to the rest of humanity. When it comes to foreign policy, Trump has two primary rhetorical modes. The first is transactional: America is being ripped off by other nations, and must cut better deals. The second is civilizational: America is part of the Judeo-Christian West, which is threatened by “radical Islam.” The former defines the world as a struggle between America and its trading partners. The latter defines the world as a struggle between civilizations., and a former enemy like Russia as a natural ally in this clash of civilizations. Neither mode suggests that promoting freedom for peoples of all nationalities, races, and religions serves America’s interest. Both are fundamentally zero-sum.^21^

Whatever the case – and history will soon teach us whether the Trump experience is just one more wave in the longer tidal movement of American history – the work of historian Richard Hofstadter may help us to put the present in the relativizing light of history. As a historian who was uncommonly au fait with social science developments of his day, he playfully drew on sociology and social psychology to fathom the darker social movements of his time. He developed a deep understanding – an empathy almost – of such larger social forces as populism, and the underlying triggers of right-wing movements as they burst on to the political scene. He was fully aware of the way that European and American social-science traditions had cross-pollinated following the exodus of European intellectuals from totalitarian persecution in Nazi-Germany, and of the new light cast upon haunting questions of totalitarianism and its supporters. Current American trends alerted him to the longer history of the dark undercurrents in American life such as the paranoid world view or the anti-intellectualism in American life - both continuing counterpoints to the Enlightenment rationalism of America’s hallowed national creed and self-image. The sociological inspiration came out most clearly in his analysis of the age of reform.^22^ There he took his cue from Max Weber who, against Karl Marx’s analysis of class and class conflict, had emphasized the importance of social status, the relative rank in society in terms of esteem and prestige that groups of people hold. As modern societies constantly undergo changes in these rank orders of status, people are subject to feelings of status anxiety and status loss. They collectively develop responses that tend to be more emotional, more in the nature of gut feeling than of rational analysis. Those are the moments when Enlightenment humanism and rationalism can no longer adequately explain the world we are living in. Max Weber, as he observed Germany’s hectic industrialization and the social turmoil it brought, already speculated that individuals, unmoored by these developments, cut loose from communal settings as these had harbored them before, could become vulnerable to a despotic leader. Rather than being open to appeals based on the rational calculation of self-interest, collective behavior in modern history provides ample evidence for the persistent power of unreason and anti-intellectualism. As Sigmund Freud warned his readers in 1915, the “primitive, savage and evil impulses of mankind have not vanished in any individual,” but are simply waiting for the opportunity to show themselves again. And when they do, they bring to the surface what Friedrich Nietzsche called “ressentiment” – “a whole tremulous realm of subterranean revenge, inexhaustible and insatiable in its outbursts.” Ressentiment - caused by an intense mix of envy, humiliation and powerlessness – is not simply the French word for resentment. In the early twentieth century, the German sociologist Max Schler developed a systematic theory of ressentiment as a distinctly modern phenomenon - ingrained in all societies where formal social equality between individuals coexists with massive differences in power, education, status, and property ownership. In an era of economic globalization, these disparities now exist everywhere, along with enlarged notions of individual aspiration and equality. Christopher Lasch, in his study of narcissism, specifically mentioned these unhinged aspirations and feelings of entitlement.^23^ These unsettling trends have affected not only internal status balances within individual societies, but also more globally the relative status of individual nations. This might allow us to examine the workings of ressentiment, and status anxieties, across varied countries and classes, as fears of economic stagnation, if not of comparative slippage, have fed into the reservoir of collective anger. It might also go a long away to account for the fact that Donald Trump, the flashy plutocrat tormented by his lowly status among Manhattan’s cultivated liberals, the perennial outsider seeking revenge upon the snobbish coteries of the established elite, is so patently allergic to any slight to his public persona. Only so can we understand him constructing a narrative of people duped by domestic party politics, jettisoned and cast aside, and of America being cheated by rival nations. Thus, he can also offer himself as representing the collective anger of his followers, although obviously coming from a world that in its ostentation flies in the face of all those he aims at representing.^24^

Trump, it may be clear, is not an isolated, purely American phenomenon. He is the American version of trends and developments more widely occurring in western democracies. For every Trump Europe can offer a homegrown equivalent, thrown up by similar reservoirs of seething anger. As in America, we see a retrenchment from an enlightenment ideal centering on the rule of law, and equal individual rights, projected on to the canvass of a trans-national Europe. Instead we see a hidebound retreat within the traditional borders of the nation and the nation-state. We see similar signs of parties and politicians out to delegitimize the prevailing democratic arrangements, at the European level and within its individual member states. They engage in disparaging the idea of representative democracy, of parliaments, the courts, the media. As members of parliament they do not shrink from calling the institution “fake,” as Geert Wilders does in the Netherlands. They promote programs of what they call “illiberal democracy,” as happens in Hungary. Anti-democratic forces are at work all over Europe, fed by resentments and anger stoked by floods of refugees, by economic uncertainty and stagnation, by alienation from the large, anonymous institutions of what calls itself the European Union. There is the return to nationalism and national borders, to dreams of national sovereignty. There is the defense of national identity against the threat of Islamization. Older repertoires, helping people define - collectively and narrowly - “who they are” have been revived, threatening a return of ethnocentrism and nativism. The air is replete with echoes a century old. Commonly they are seen as the voice of populism. We may also see them as so many signs of fascism rising.

It is good, in conclusion, to remind ourselves of what we may be losing by going back in our minds to President Obama’s Farewell Address. As journalist Charles Blow put it in the *New York Times* (January 11, 2017): “(L)istening to the president’s farewell address, I was hit with the force of a brawler that the decency and dignity, the solemnity and splendor, the loftiness and literacy that Obama brought to the office was extraordinary and anomalous, the kind of thing that each generation may only hope to have in a president.” In light of everything I have argued so far, we should also remind ourselves how hard it is to fully take the measure of Obama’s achievement as president without taking into account the continued undercutting of his every policy by the combined destructive force of the Republicans in Congress (who may have seen it as their overarching policy goal to obstruct anything coming from Obama’s Democrats). Obama has been up, for the full duration of his two-term presidency, against precisely the obstructionist forces undermining America’s democracy that we have discussed here.

## Footnotes

1
http://edition.cnn.com/2016/03/03/politics/mitt-romney-presidential-race-speech/

2 Jonathan Raban: *New York Times*, 7 Januari 2017, Sunday Review. Raban is the author of *Surveillance*, a gripping dystopian fantasy set in the Seattle area, in which he evokes a police state nightmare in an Orwellian genre that will occupy us shortly. (Jonathan Raban, *Surveillance* (New York: Vintage Books, 2006)).

3 Rosario Forlenza, Bjørn Thomassen, “Decoding Donald Trump: The Triumph of Trickster Politics,” http://www.publicseminar.org/2016/04/decoding-donald-trump-the-triumph-of-trickster-politics/#.VzHK2oR97ct

4 C.G. Jung: „Zur Psychologie der Schelmenfigur,” in: Paul Radin, Karl Kerényi, C. G. Jung, eds. *Der göttliche Schelm. Ein indianischer Mythen-Zyklus. Schelmen-Zyklus*, translated by Ilse Krämer, (Rhein-Verlag, Zürich 1954). Claude Lévi-Strauss: „The Structural Study of Myth,” *The Journal of American Folklore* 68/270, October – December 1955, pp. 428–444.

5 Michael Andrew Y. Sakamoto*, An Empty Room: Butoh Performance and the Social Body in Crisis*. (University of California, Los Angeles 2012, p.191).

6 The leader of the Republican Party in the Senate, Mitch McConnell, is on record declaring this mission for the Republicans in Congress. *The Nation* and *The Undercurrent* obtained an audio recording of Mitch McConnell’s remarks to a secret strategy conference of conservative millionaire and billionaire donors hosted by the Koch brothers, called “American Courage: Our Commitment to a Free Society.” In the question-and-answer period following his June 15 session titled “Free Speech: Defending First Amendment Rights,” McConnell said: “So in the House and Senate, we own the budget. So what does that mean? That means that we can pass the spending bill. And I assure you that in the spending bill, we will be pushing back against this bureaucracy by doing what’s called placing riders in the bill. No money can be spent to do this or to do that. We’re going to go after them on healthcare, on financial services, on the Environmental Protection Agency, across the board [inaudible]. All across the federal government, we’re going to go after it.” Notice the Orwellian Newspeak twist where words like Free Society and Free Speech have come to mean their opposite.
https://www.thenation.com/article/caught-tape-what-mitch-mcconnell-complained-about-roomful-billionaires-exclusive/

7 YouGov as quoted by Joffe: http://www.zeit.de/2016/45/donald-trump-aufstieg-direkte-demokratie/seite-3

8
http://www.thedailybeast.com/articles/2016/08/22/steve-bannon-trump-s-top-guy-told-me-he-was-a-leninist.html

9 This is not the place for a discussion in depth of fascism as a historical phenomenon. For a recent selection of critical essays on the subject, see: *Constellations*, Vol. 15, issue 3, September 2008.

10 Diggins, John P., “Mussolini as American Hero.” *Mussolini and Fascism: The View from America*, 58–74. Princeton University Press, 1972. http://www.jstor.org/stable/j.ctt13x16kq.9. Gian Giacomo Migone, *The United States and Fascist Italy: The Rise of American Finance in Europe* (translated from the Italian and with a preface by Molly Tambor) Cambridge University Press.

11 Robert Michels, “Wirtschaftliche und politische Betrachtungen zur alten und neuen Welt (Ita;lien und die Vereinigten Staaten von Nordamerika) *Kölner Vorträge* III. Band/2. Heft (Leipzig: G.A. Gloeckner, 1928)*.*

12 Karl Mannheim, *Diagnosis of Our Time: Wartime Essays of a Sociologist* (London: Kegan Paul, Trench, Trubner & Co., 1943) The book appeared in a series famously started by Mannheim himself: “International Library of Sociology and Social Reconstruction.”

13 Jonah Goldberg*, Liberal Fascism: The Secret History of the American Left, From Mussolini to the Politics of Meaning*.

14 Dorothy Thompson, in: Helen Thomas, *Watchdogs of Democracy? The Waning Washington Press Corps and How It Has Failed the Public* (New York: Scribner, 2006) p. 172.

15 Philip Roth, *The Plot Against America*. (Boston, New York: Houghton Mifflin, 2004).

16 Trump told supporters: “Would I approve waterboarding? You bet your ass I would. In a heartbeat. I would approve more than that. It works.” (https://www.theguardian.com/us-news/2015/nov/24/donald-trump-on-waterboarding-even-if-it-doesnt-work-they-deserve-it; in November he re-iterated his support, after having backtracked at first. “Donald Trump offered renewed support on Tuesday for the use of torture while repeatedly comparing a proposed trans-Pacific free trade agreement to rape. https://www.theguardian.com/us-news/2016/jun/28/donald-trump-ohio-rally-isis-torture-tpp-rape.

17 Alain Frachon, “L’émergence du ‘trumpisme’,” *Le Monde, Sélection hebdomadaire* (Saturday, 19 March, 2016) p.12.

18 Richard Hofstadter, “The Pseudo-Conservative Revolt (1955),” in: Daniel Bell, ed., *The Radical Right* (New York: Doubleday, 1964; p. 95.

19 It doesn’t take a macho-populist in the Trump vein to speak of carnage when surveying the contemporary American landscape. Left-wing journalist Chris Hedges, in an interview with the French newspaper *Le Monde*, on the classic Tocquevillean theme of democracy in the United States (which Hedges calls nothing more than a faҫade) he has this to say: “Drive across the United States, what you see is wreckage and ruin.” As he sees it, this is the problem of declining empires. “They extend beyond their capacities and end up collapsing from the inside.” Precisely this sense of an empire ending, of America slipping down the global charts, is one factor behind the rage of the insurgents in the U.S. (*Le monde*, Saturday, January 7, 2017, p. 10).

20 Shaun King*, New York Daily News*, Monday, November 21, 2016: “At an Alt-Right conference being held, of all places, in a federal building in Washington D.C., Spencer didn’t even attempt to hide his bigotry and anti-Semitism. Richard Spencer, who coined the term “alt-right” and is seen as one of its founders and public intellectuals, openly wore his Neo-Nazi heart on his sleeve. According to the NY Times, he railed against Jews and, with a smile, quoted Nazi propaganda in the original German. America, he said, belonged to white people, whom he called the “children of the sun,” a race of conquerors and creators who had been marginalized but now, in the era of President-elect Donald J. Trump, were “awakening to their own identity.” As he finished, several audience members had their arms outstretched in a Nazi salute. When Mr. Spencer, or perhaps another person standing near him at the front of the room — it was not clear who — shouted, “Heil the people! Heil victory,” the room shouted it back.”

21 On this see Peter Beinart: https://www.theatlantic.com/international/archive/2017/01/trumps-new-foreign-policy-direction/513614/?utm_source=polfb&utm_source=nl-politics-daily-011917

22 Richard Hofstadter, *The Age of Reform: From Bryan to F.D.R.* (New York: Alfred A. Knopf, 1961) Esp. chapter IV: “The Status Revolution and Progressive Leaders”.

23 Christopher Lasch, *The Culture of Narcissism: American Life in an Age of Diminishing Expectations* (New York: W.W. Norton, 1979).

24 How it must have stung Trump to read these words from Philip Roth: ““I was born in 1933,” Roth wrote, “the year that F.D.R. was inaugurated. He was president until I was 12-years-old. I’ve been a Roosevelt Democrat ever since. I found much that was alarming about being a citizen during the tenures of Richard Nixon and George W. Bush. But, whatever I may have seen as their limitations of character or intellect, neither was anything like as humanly impoverished as Trump is: ignorant of government, of history, of science, of philosophy, of art, incapable of expressing or recognizing subtlety or nuance, destitute of all decency, and wielding a vocabulary of seventy-seven words that is better called Jerkish than English.”
http://www.haaretz.com/us-news/.premium-1.766903?utm_content=%2Fus-news%2F.premium-1.766903&utm_medium=email&utm_source=smartfocus&utm_campaign=newsletter-daily&utm_term=20170123-11%3A01

